# “*There is no reward penny for going out and picking up youths*”: issues in the design of accessible youth healthcare services in rural northern Sweden

**DOI:** 10.1186/s13104-019-4108-4

**Published:** 2019-02-04

**Authors:** Cecilia Hultstrand Ahlin, Dean Carson, Isabel Goicolea

**Affiliations:** 1 Department of Nursing, Umeå University, 901 85 Umeå, Sweden; 2 Northern Institute, Charles Darwin University, Casuarina, NT 0909 Australia; 3 Unit of Epidemiology and Global Health, Department of Clinical Medicine and Public Health, Umeå University, 901 87 Umeå, Sweden

**Keywords:** Rural, Youth, Health services, Youth clinics

## Abstract

**Objective:**

There is a continuing challenge to ensure equitable access to youth healthcare services in small rural communities. Sweden’s ‘youth clinic’ system is an attempt to provide comprehensive youth health services from a single centre, but many small rural communities have not adopted the youth clinic model. This study uses one case study to examine what the issues might be in establishing a youth clinic in a small rural community. The objective of this paper is to examine the issues around youth healthcare access in one municipality without a youth clinic, and to explore whether and how a youth clinic model might contribute to access in this municipality.

**Results:**

Three categories emerged from the analysis; (i) rural closeness; both good and bad, (ii) youth are not in the centre of the healthcare organization, and (iii) adapting youth clinics to a rural setting. While limited to one case example, the study provides valuable insights into youth health service planning in particular types of rural communities. This paper identified structural barriers to developing youth-specific services, and some alternative approaches that might be more suitable to smaller communities.

**Electronic supplementary material:**

The online version of this article (10.1186/s13104-019-4108-4) contains supplementary material, which is available to authorized users.

## Introduction

Even though youth is a relatively healthy period, it is a time when risks of morbidity associated with sexual health related problems and violence may arise [1–4]. Since behaviours that are adopted during this period are likely to last throughout life, youth is also a period of many opportunities [2, 3]. Responding to youths’ health needs is however challenging since their needs are diverse, and their access to services is poorer than for children and adults [5]. Sweden’s principal response to the challenges of delivering healthcare services to youth has been the development of specialist youth clinics (YCs) aimed at people aged 13–24 years. There are currently approximately 300 YCs striving to promote physical and psychosocial health with a special focus on sexual and reproductive health and responding to youths’ healthcare needs. YCs are often located outside general healthcare facilitates and are staffed with midwifes, counsellors and physicians, however, the size, number of staff and variety of profession varies widely between YCs [6, 7]. However, many rural municipalities in northern Sweden do not have YCs, and there has been no research into how youth health needs are managed in these areas. The aim of this paper is to examine the issues around youth healthcare access in one municipality without a YC, and to explore whether and how a youth clinic model might contribute to access in this municipality. While limited to one case example, the study provides valuable insights into developing research into youth health services and access in small rural communities, and considering alternatives to the ‘clinic’ approach which generally seems more suitable to larger rural/regional communities.

Strategies to improve the accessibility of healthcare services for young people range from making changes within existing healthcare services, developing differentiated services only for youth, and peer-education programs that bring health information and care to the spaces where youth are [2, 8]. Despite these efforts, research shows that healthcare services remain, in general, less accessible for young people than for adults in many countries [2, 9]. Furthermore, rural youths often experience scarce support [10] and a deficit of guidance [11], plus, little attention is given to youths’ wishes in policy development [12].

Rural health is a complex issue [13], according to studies primarily from North America and Australia, rural youth experience poor access to healthcare services compared to urban youth [14]. Lack of anonymity, a rural culture of self-reliance, and stigma have been described as barriers to seeking mental healthcare among rural youth, and studies highlight that solutions designed for adults do not always work for young people [8]. Research also shows, however, that well designed rural youth health services foster close relationships between youth and health professionals, resulting in more personalised and continuing models of care. Youth health services are also more likely to successfully embrace Internet and other technologies which can at least in part deal with issues of isolation and lack of local services [15]. In Sweden, a country where 2% of youths live in rural areas, rural youth health studies are scarce with the exception of some consideration of (rural and urban) Sámi youth health status [16].

## Main text

### Methods

This study was conducted in a rural municipality in northern Sweden (see Table 1 for context information), where youth comprise about 13% of the population. Local health services include two small rural hospitals, elderly care and some limited allied health facilities. The municipality has been proactive in adopting eHealth strategies, including having Sweden’s first ‘virtual health room’ which uses distance bridging technologies to provide basic health services [17]. The municipality is typical of rural northern Sweden in terms of small population, isolation from larger services, and challenges in designing health services that are sustainable financially and can attract well qualified health professionals [18].

**Table 1:** Demographic and geographic characteristics of the municipality

Area	7500 km^2^
Number of inhabitants, 2016	6000
Median age	47.1
Number of people aged 13–24, 2016	750
Density of inhabitants	0.8/km^2^
Distance to provincial capital	300 km

Healthcare services that are available in the rural municipality are provided by one facility, the local petit hospital. This hospital is organized as a family physician led hospital and offers comprehensive general healthcare, delivered by approximately 30 healthcare professionals with different competences.

The research involved six interviews with stakeholders and one focus group discussion (FGD) with seven young people (see Table 2). Stakeholders were purposively recruited with the help from a local gate-keeper. The criteria for recruiting participants for the interviews were having lived in the municipality for the last years, were in contact with youths and/or healthcare in their profession. Potential participants were first contacted by email and afterwards information was sent to them including a written invitation.

**Table 2:** Characteristics of participants

Participants in individual interviews		Participants in focus group discussion	
Occupation	Sex	Occupation	Sex
School counsellor	Woman	Student	Girl
School nurse	Woman	Student	Girl
Nurse	Woman	Student	Girl
Expertise in rural medicine	Man	Student	Girl
Expertise in rural health	Man	Student	Boy
Social worker	Woman	Student	Boy
–	–	Student	Boy

Young participants in the FGD were randomly recruited by another gate-keeper who worked at one of the schools in the municipality. The criteria were being over 16 years old and living in the municipality. This gate-keeper organized the FGD by inviting youths to participate.

The research team developed two interview guides one for the individual interviews (see Additional file 1), and one for the FGD (see Additional file 2). The interviews followed an emergent design, meaning that relevant topics that emerged during one interview were included in the following one. The interview guides were based on broad open ended questions to enable participants to speak freely and answer our questions with their own words about: (a) local youth healthcare needs, and the impact of ‘being rural’ on needs and access, (b) facilitators of, and barriers to healthcare access for youth, (c) local service ability to meet the needs of youth, and service gaps, and (d) collaboration between the healthcare organization, school, social workers and other institutions. The interviews lasted between 28 and 64 min, they were verbatim transcribed and analysed using qualitative content analysis [19]. Coding was performed by CHA with continuous discussion with IG. The transcripts were coded line by line, and afterwards codes were grouped together to develop categories. Categories were then discussed and modified among all authors, through an abductive process of going back and forth from the categories to the text, and discussing between all the authors. Ethical approval was granted by Umeå University Ethical Review Board (Dnr. 2015-190-31Ö) and written informed consent was obtained from each participant.

### Results

Three categories were developed from the analysis; (i) rural closeness; both good and bad, (ii) youth are not in the centre of the healthcare organization, and (iii) adapting youth clinics to a rural setting. The categories point out both structural barriers to developing youth-specific services in rural settings, as well as some alternative approaches that might be more suitable to smaller communities.

#### Rural ‘closeness’ as both facilitator and barrier

Sense of community, solidarity and togetherness were features that youth valued highly from the place where they lived. This was also reflected in the trust they had in the different health professionals they met. Long term relationships with health professionals was seen as a positive thing.

*“Sometimes it is an incredible strength to go to a district physician that I have known since many years back, he knows my family, it is a strength”*. (Interview 1)

Closeness in the rural setting was linked with personal commitment, pushing professionals to “do more”; with the example given of buying emergency contraception when the young person could not afford it.

*“There was one that needed emergency contraceptive, but could not afford it, so she went to the school counsellor so that she could buy it for her”* (Youth, focus group discussion)

Lack of anonymity and a sense of insufficient privacy and confidentiality was considered as characteristic of rural small settings and as especially challenging for youth, particularly when it came to mental health problems, sex and relationships, drugs and counselling.

*“When it comes to youth and birth control pills, abortion, or chlamydia I think some manage, but for some it is not easy to go to [the community hospital] and ask for the*-*day*-*after*-*pill if your friend’s mother works there”*. (Interview 2)

Close relationships enabled and demanded cooperation between different health professionals and service providers. Referrals between local providers were seen as generally well managed.

“*We have quite much collaboration, well, some overall collaboration groups, but we also have everything when it’s necessary, with individual cases we have pretty good collaboration*”. (Interview 3)

However, local providers were not well engaged with professional networks outside of the municipality. The small number of local providers and the isolation from external expertise meant that building or extending skills in youth health work was very difficult.

#### Youth not being a primary client group for healthcare organizations

Besides the hospitals, the schools had health service resources in the form of school nurses and school counsellors, although these were usually shared between a number of schools. Furthermore, these resources were not perceived to be well integrated in the healthcare organization.

The community hospital was perceived as a good place where everyone could get access to many different health services. However, the requirement to book appointments was considered a barrier to access, with ‘drop in’ services seen as more suitable because of difficulties in organising transport and managing other time commitments. However services with drop-in was explained to be missing. As one informant put it:

*“The social services have no drop*-*in, the healthcare has no drop*-*in, but well, I guess the school curator is available if you drop by and check if she is there, but they are at some different schools so they are not always at the same schools”*. (Interview 3)

General health services placed a high priority on elderly care and the health needs of a relatively old population, with lower priority given to preventative care, and even lower priority to youth health. As one informant put it:

*“There is no reward penny for going out and picking up youths”*. (Interview 1)

Despite investments in eHealth services in the municipality, there had been little consideration of how these might be used to improve youth access.

#### Adapting youth clinic models to rural settings

Participants reflected on how the Swedish model of youth clinics could be revisited to better meet the needs of youth in a rural municipality.

*“It does not have to be the healthcare organization that would operate a function like that. Instead, maybe one could sit down at the youth recreation centre, maybe the school health team could be engaged and sit down in the school halls”*. (Interview 1)

A youth clinic might operate differently in a rural area. For example, it was suggested that a drop in open clinic held by various health professionals one night per week or per month might be beneficial. The employment of a so called “youth officer” who would work only with youth was also suggested as a possible improvement. Participants considered that services for youth should integrate diverse professionals and that both promotive, preventive and in some case treatment services should be offered. The aim should not be the “care” per se, but to empower youths.

“*Information, information, information. Knowledge, making our youth wise. Make them independent so that they dare to make their own choices*”. (Interview 1)

An alternative view was that youth health services could simply be better integrated within the existing general health services, rather than needing to establish separate youth services.

*“I think that the health centre are following people throughout life. It is not possible to have specific clinics, you are supposed to follow people through their lives with their ailments they have”*. (Interview 6)

The small youth population was mentioned as the main reason making it difficult to justify a youth clinic in a rural setting. Specialized services generally were not considered workable in rural areas because they are financially unsustainable.

### Discussion

The aim of this paper was to examine the issues around youth healthcare access in one municipality without a youth clinic, and to explore whether and how a youth clinic model might contribute to access in this municipality. Rural youth in this study experienced similar advantages and disadvantages when it came to accessing health services as have been found in studies elsewhere [2, 10, 14, 20, 21]. Youth can have positive close relationships with providers, but they also worry about lack of privacy. Youth were also found to be marginalised in the general healthcare system, meaning little attention had been paid to designing youth friendly services. Nevertheless, some form of youth clinic, and better adaptation of eHealth services for youth were considered possibilities for improving service access.

Youth health is in general not seen as high priority in Sweden [22] and elsewhere [2, 23], since there are perceived to be few youth and the highest priority is elderly care. This is not surprising since both in rural policies and research, youth health is not prioritized [2, 22, 23]. The (perceived) small number of youth who live in the area was also considered to be the main barrier for creating a youth specific service. However, the possibility of a YC adapted to local needs was considered by some stakeholders as a future development, in fact YCs are already quite heterogeneous in the way they work depending on their location [7, 22]. On the other hand, some stakeholders felt that the YC model was inappropriate for this setting, where key model attributes such as location, staffing and management would need to be different to the ‘standard’. In any case, specialised youth services would always be at risk of financial challenges.

The existing model of youth healthcare service provision in this municipality benefited somewhat from close relationships between local service providers, although there were opportunities identified to improve coordination between community based and school based services. Service provision was weakened by the lack of strong relationships between local providers, and external (usually specialist) providers. The lack of relationships with these specialists meant that there was limited potential to build local expertise in youth health.

### Conclusion

Prioritising youth health is likely to continue to be a challenge in small rural municipalities whose demographic profile is dominated by elderly people. There may be a need for local youth health ‘champions’ to promote better attention to youth service design and delivery. Even so, small youth population numbers, and high costs of delivering specialised services in these contexts mean that the simple adopting of the youth clinic model which have been successful in other parts of Sweden may not be possible. Rather, local solutions that account for the small client population, large geographic area and the limitations and opportunities presented by the configuration of existing health services need to be developed. While this research identified some possibility for ‘partial’ solutions such as part-time youth clinics, there is a strong need for further research in what models of youth healthcare might be developed in these contexts.

## Limitations

Our study has some limitations. Firstly, our study is limited to a single case study with a small sample size. However, a single case study can provide insights into more extended future or comparative work. Secondly, we have had limited engagement with the diversity of youth living in the region, and limited engagement with service providers based outside of the region. For example, we might have failed to include the perspectives of young people who are out of school or study in the city, which might have different healthcare needs and consequently, different perception of the existing services. It is therefore possible that our results could have been further developed if we incorporated greater diversity into our sample. Nevertheless, it provides some important insights into the environment around youth health services in a small rural community.

## Additional files

**Additional file 1.** Interview guide for individual interviews. The file includes the questions guiding the semi-structured interviews used to obtain the qualitative data analysed in this paper.

**Additional file 2.** Interview guide for focus group discussion. The file includes the questions guiding the semi-structured focus group discussion used to obtain the qualitative data analysed in this paper.

## Abbreviations

YC: youth clinic
FGD: focus group discussion
