# Supplementary material for: “There is no reward penny for going out and picking up youths”: issues in the design of accessible youth healthcare services in rural northern Sweden
Source: BMC Res Notes. 2019 Feb 4;12:74. doi: 10.1186/s13104-019-4108-4 (PMC6360772; doi:10.1186/s13104-019-4108-4)
Supplement: Supplementary file 1 — Additional file 1. Interview guide for individual interviews. The file includes the questions guiding the semi-structured interviews used to obtain the qualitative data analysed in this paper. [file 13104_2019_4108_MOESM1_ESM.docx]

**Additional file 1**

Interview guide; individual interviews

1. Can you talk a little bit about your background and describe your work for me, what are your tasks?
2. How does the collaboration with other professions look like?
3. What do you think is the foremost strength with how the organization here is organized?
4. What do you think is the biggest weakness with the organization?
5. Form your perspective, what health needs do the youths here in XX have?
6. How do you work to meet these needs, are there any certain strategies or guidelines?
7. How would you describe youths’ access to care?
8. What kind of healthcare and health services are offered here?
9. Is there any service that that is not offered here?
10. What obstacles are youths facing when they seek care, are there any barriers?
11. What do you think is the main reason for youths to seek care?
12. How would you describe XX as a place to live and grow up in?
13. From a social perspective, how would you describe the surrounding environment?
14. We have talked about youths’ health needs, how do you evaluate your ability to meet these needs?
15. Do you offer any other service to compensate for the lack of a YC?
16. What role has the school, in terms of providing health services?
17. What effect on youths’ health and well-being do you think a YC would have here?
18. Do you think there is need to implement a YC here?
19. From your perspective, what is the most important thing to improve here?
