# Supplementary material for: “There is no reward penny for going out and picking up youths”: issues in the design of accessible youth healthcare services in rural northern Sweden
Source: BMC Res Notes. 2019 Feb 4;12:74. doi: 10.1186/s13104-019-4108-4 (PMC6360772; doi:10.1186/s13104-019-4108-4)
Supplement: Supplementary file 2 — Additional file 2. Interview guide for focus group discussion. The file includes the questions guiding the semi-structured focus group discussion used to obtain the qualitative data analysed in this paper. [file 13104_2019_4108_MOESM2_ESM.docx]

**Additional file 2**

Interview guide; focus group discussion

1. Can you describe how it is to grow up and live here in XX?
2. What are the benefits with living here?
3. What are the disadvantages with living here?
4. What do you usually do on your spare time?
5. If you were supposed to describe this place with three words, what words would it be?
6. Can you describe how it is to make new friends here?
7. Do you often visit the school nurse? Why? Why not?
8. Do you often visit the school counsellor? Why? Why not?
9. Is there something you feel that you cannot talk with the school nurse/counsellor about? If yes, what and why?
10. If you seek care from someplace else, where do you go and how do you do?
11. Are there any difficulties with seeking healthcare?
12. Have you heard about YCs? If yes, what are your thoughts about them?
13. How would you feel if a YC were implemented here?
14. What is the least favourable thing with this municipality?
15. What is the best thing with this municipality?
